# Towards a Latin American neuropsychiatry: challenges and opportunities

**DOI:** 10.1016/j.lana.2025.101322

**Published:** 2025-12-02

**Authors:** Jesús Ramírez Bermúdez, Sheila Castro-Suarez, Luciana D'Alessio, Jorge Holguín Lew, Louise Makarem Oliveira, Mônica Sanches Yassuda, Hernando Santamaría-García, Andrea Slachevsky, William Tamayo Agudelo, Julio Torales, Norha Vera San Juan, Vaughan Bell

**Affiliations:** a National Institute of Neurology and Neurosurgery, México City, Mexico; b Centro Básico de Investigación en Demencia y Enfermedades Desmielinizantes del Sistema Nervioso, Instituto Nacional de Ciencias Neurológica, Lima, Perú; c University of Buenos Aires, Institute of Cellular Biology and Neurosciences (CONICET), Faculty of Medicine, Argentina; d Department of Psychiatry, University CES, Medellín, Colombia; e Department of Neurology, School of Medicine of Ribeirão Preto, University of São Paulo, Brazil; f Department of Gerontology, School of Arts, Sciences and Humanities, University of São Paulo, São Paulo, Brazil; g PhD Program of Neuroscience, Pontificia Universidad Javeriana, Bogotá, Colombia; h Centre of Memory of Cognition Intellectus, University Hospital San Ignacio, Bogotá, Colombia; i Neuropsychology and Clinical Neuroscience Laboratory (LANNEC), Physiopathology Program – Institute of Biomedical Sciences (ICBM), Neuroscience and East Neuroscience Departments, Faculty of Medicine, University of Chile, Santiago, Chile; j Geroscience Center for Brain Health and Metabolism (GERO), Santiago, Chile; k Memory and Neuropsychiatric Center (CMYN) Neurology Department, Hospital del Salvador & Faculty of Medicine, University of Chile, Chile; l Servicio de Neurología, Departamento de Medicina, Clínica Alemana-Universidad del Desarrollo, Santiago, Chile; m Cooperative University of Colombia, Medellín, Colombia; n Universidad Nacional de Asunción, Facultad de Ciencias Médicas, Cátedra de Psiquiatría, San Lorenzo, Paraguay; o Universidad Nacional de Caaguazú, Instituto de Regional de Investigación en Salud, Coronel Oviedo, Paraguay; p Universidad Sudamericana, Facultad de Ciencias de la Salud, Salto del Guairá, Paraguay; q Institute for Global Health, University College London, London, UK; r Clinical, Educational and Health Psychology, University College London, UK; s Department of Neuropsychiatry, Maudsley Hospital, London, UK

**Keywords:** Psychiatry, Neurology, Psychology, South America, Latin America, Neuropsychiatry

## Abstract

From the impact of armed conflict and political violence to the neuropsychiatric consequences of neglected tropical diseases, Latin America has a unique profile of region-specific risk factors that mean it is not always well-served by neuropsychiatric practice developed in high-income regions. Here, we review the region-specific neuropsychiatric characteristics of traumatic brain injury, stroke, epilepsy, dementia, functional neurological disorder, infectious diseases, environmental health risks, and substance use. Additionally, we identify structural challenges for neuropsychiatric health and suggest pathways to develop a specifically Latin American neuropsychiatry as a cross-disciplinary, multi-professional field based on practical steps to strengthen research capacity, training, clinical practice, and care delivery. Latin America should be a priority for neuropsychiatry, and we argue for a Latin American neuropsychiatry that has much to offer the region and much to contribute worldwide.

## Introduction

Neuropsychiatry treats, prevents, and researches problems at the interface of psychiatry and neurology.^1^ It is often considered a sister discipline to behavioural neurology, as it focuses on managing the psychiatric complications of neurological disorders alongside conditions that require crossover expertise in psychiatric and neurological fields, such as functional neurological disorder, tic disorders, neurodegenerative disorders, delirium, and the impact of substance use. Unfortunately, neuropsychiatric patients frequently fall between specialities, meaning traditional services are often poorly prepared to address their needs and specialist knowledge among clinicians is rare.

Latin America should be a priority region for neuropsychiatry. Health challenges in the Latin American region—defined here as Spanish and Portuguese-speaking countries in South America and the Caribbean—frequently cross the mental and neurological health divide.^2^ This is due to complex risk factors that arise from its marked income inequality within and between countries, uniquely varied geography, endemic poverty, and complex social history.^3^ Although Latin America is an economically diverse region, the overall level of development classifies it as a region in the ‘Global South’ with significant economic and development challenges remaining even within higher-income countries in the region, particularly in the area of health.^4^ From the consequences of armed conflict and political violence, to the neurological effects of neglected tropical disease, it has a unique profile of region-specific challenges that are poorly served by neuropsychiatric practice developed in, and for, other regions. It also offers valuable resources, including a pool of diverse health professionals and dedicated research and training centres, although both remain unevenly distributed and are often concentrated in relatively few urban centres.

Unfortunately, much of the evidence base for neuropsychiatric practice is drawn from Western countries and largely from English-language sources, meaning it frequently fails to address regional priorities and fails to include research that does.^5^ Indeed, medical prediction models developed in high-income countries perform poorly in Latin America^6^^,^^7^ illustrating that evidence cannot simply be ‘imported’ and applied. In terms of clinical care, some professionals have strong local traditions of neuropsychiatric training and practice, while others remain isolated, making it challenging to train, collaborate, and lobby for region-wide improvements in practice and funding.

In this Personal View, we reviewed the literature to identify what distinguishes neuropsychiatric needs in Latin America based on the evidence and epidemiology for key disorders. We also outline a vision for a Latin American neuropsychiatry focused on regional priorities, supported by a multi-professional network of mutual support, training, and research.

## Major neuropsychiatric causes and conditions in Latin America

**Fig. 1:**
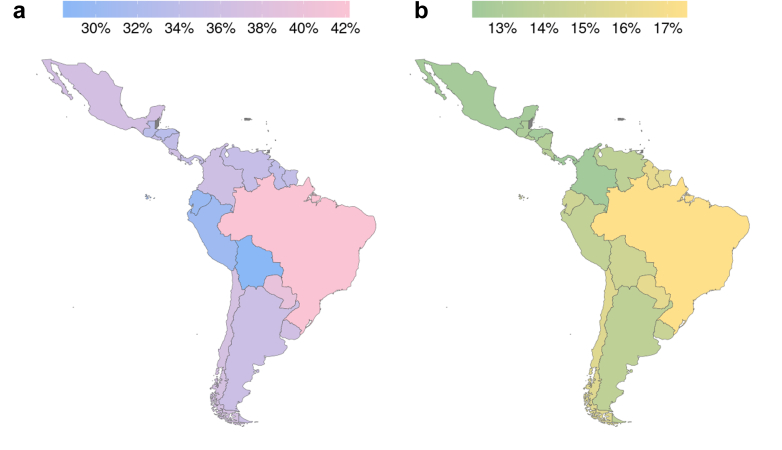
Global Burden of Disease Study 2019 prevalence of a) neurological disorders and b) mental disorders in Latin America.

Neurological and mental disorders have a lifetime prevalence of 28–42% and 12–17% respectively in Latin America (Fig. 1), and co-occurrence is common, with high rates of co-morbidity and resultant disability.^8^

### Traumatic brain injury (TBI)

Neuropsychiatric complications are common following traumatic brain injury and can include depression, anxiety, personality changes, apathy, impulsivity or aggressive behaviour, impaired memory, and sleep disturbances.^9^ Latin America is often described as having world-leading rates of TBI,^10^ but the true incidence and prevalence remain unclear. Regional estimates range from globally low rates,^11^ to globally average rates,^12^ to globally leading rates of TBI.^10^ However, all sources raise significant concerns about the poor quality of data from the region and report substantial inter-country variability. Given that Latin America has some of the highest years-of-life-lost (YLL) to injury more broadly,^13^ it is likely that TBI is a substantial contributor to disability. Traffic accidents,^14^ work-related injuries^15^ and violence^16^ are major causes of traumatic brain injury with the latter cause particularly distinguishing Latin America from other regions. Only a handful of studies have looked at post-TBI neuropsychiatric outcomes in the region. In contrast to the meta-analytically estimated prevalence rates of depression (35.35%; 95% CI 24.64–46.87) and anxiety (28.64%; 95% CI 17.99–40.65) reported after TBI in non-Latin Global South countries,^17^ Cuba has reported elevated rates of post-TBI anxiety (48%),^18^ and Colombia has documented increased rates of post-TBI depression (40%),^19^ although these are small, opportunistic studies and the quality of evidence remains poor. Notably, Latin American studies appear to more frequently focus on the family after TBI than on individual patients, likely reflecting the fact that the family is often the only option for post-TBI support, as well as the importance of *familismo*—cultural values and practices that centre commitment to family well-being and unity.^20^ Although identification with *familismo* values has been associated with better family coping after TBI in Colombia and Mexico,^21^ differences in values, State support, and the economic position of families in Latin America compared to high income regions indicate that family interventions may need to be culturally specific to adequately support post-TBI family-based care, potentially adapting to include involvement of the extended family, collective decision-making, and balancing family obligations with healthcare needs.

### Stroke

Stroke is a syndrome of acute, focal neurological deficit caused by vascular infarction or haemorrhage affecting the central nervous system. Overall stroke incidence and prevalence rates are similar between Latin America and high-income regions, although the balance of stroke types is different: there is a lower proportion of ischemic stroke compared to high-income countries but a higher proportion of intracerebral and subarachnoid haemorrhage.^22^ A recent international prevalence study of post-stroke depression estimated a 17.7% (95% CI 15.65–20.0) global point prevalence of depression in stroke patients. However, only two studies from Latin America were included, both from Brazil, which reported higher prevalence rates of 28.8%^23^ and 31%^24^ respectively. Subsequent studies from Latin America have reported higher estimates of depression after stroke compared to global figures (e.g. Brazil, 26.7%^25^; Mexico 56%^26^; Argentina, 50%^27^). For post-stroke anxiety, Knapp et al.^28^ reported meta-analytic estimates of 18.7% (95% CI 12.5–24.9) for interview-measure-estimated anxiety, and 24.2% (95% CI 21.5–26.9) for rating scale estimated anxiety. Only one study from Latin America was included in this meta-analysis, a study from Brazil using a rating scale, reporting a 24% prevalence of anxiety in line with the global average. One subsequent study,^25^ also from Brazil, reported a higher than average estimate of 23.3% from an interview measure study. In terms of psychosis, a recent individual participant level meta-analysis of combined stroke and psychosis prevalence in the UK, USA, Chile and Colombia,^29^ show high rates of probable psychosis in the stroke in the Colombian population but was not able to reach any definite conclusions about regional variations due to differences in measurement methods.

### Epilepsy

Epilepsy is a neurological disorder characterised by recurrent seizures and its neurobiological, cognitive, psychological, and social consequences. The management of epilepsy has advanced considerably in Latin America but there are significant differences in its aetiology and presentation compared to high-income regions. Years lived with disability due to epilepsy reduced by 20% in Latin America from 1990 to 2019, but the prevalence of idiopathic epilepsy has not changed and there has been an increase in prevalence of secondary epilepsy syndromes.^30^ There are likely multiple factors that explain the high rates of secondary epilepsy syndromes, including high rates of neurological disorder more broadly, however, neuroinfection has been consistently identified as an important contributory factor.^31^ A meta-analysis by Alva-Díaz and colleagues estimated a neurocysticercosis prevalence of 17.37% in people living with epilepsy in Latin America.^32^ This compares to a prevalence of 2.1% in similar patients in the United States.^33^ The incidence of epilepsy is higher in rural areas of Latin America,^34^ with large studies conducted in rural areas of Bolivia,^35^ Mexico,^36^ Honduras^37^ and Ecuador^38^ reporting poor access to treatment, high levels of stigma, and/or frequent appeal to folk beliefs for causal explanation and treatment. It is unclear to what extent the rate of neuropsychiatric complications differs in patients with epilepsy in Latin America. A recent international meta-analysis of anxiety and depressive disorders in people with epilepsy, including four studies from Brazil and two from Cuba, reported no moderation by region,^39^ suggesting international rates (about 1 in 5 patients with epilepsy having comorbid anxiety and/or depression) may be reasonable estimates although caveats regarding the low number of studies from the region apply. Epilepsy may, however, be a complicating factor in the most acutely unwell psychiatric patients. A study of all patients admitted to psychiatric hospitals in Brazil's public health system reported a risk ratio of 5.7 for mortality due to idiopathic epilepsy.^40^

### Dementia

Dementia is a progressive neurological disorder characterized by a decline in cognitive function caused by various underlying conditions, such as Alzheimer's disease, vascular dementia, frontotemporal degeneration and Parkinson's disease. Compared to high-income regions, Latin America has higher rates of dementia and increasing, rather than decreasing, prevalence.^41^ The risk factors for dementia have been well characterised in high-income countries but Latin American researchers have expressed concerns that they may not fully translate to the region due to important sociodemographic differences.^42^ Indeed, compared to high-income regions, risk factors associated with disparities in social status and health account for unhealthy ageing to a far greater degree in Latin America than other regions.^2^ Although likely contributing to the higher prevalence, this also indicates a better potential for dementia prevention in Latin America.^43^ Studies from the 10/66 Dementia Research Group on dementia on low and middle income countries have reported that loss of insight for dementia symptoms (anosognosia) is differentially associated with psychiatric symptoms, education, dementia severity and socioeconomic status in Latin America compared to India and China^44^ indicating sociocultural factors are likely to be important influences on causal risk factors and presentation. However, dementia is one of the better-addressed neuropsychiatric disorders in the region. Successful networks of researchers and clinicians have been established, frameworks for regional action plans are being developed, numerous studies on interventions for caregivers of people with dementia have been conducted while standardising diagnosis, epidemiological studies, training and facilities have been cited as key goals for improving dementia care.^42^

### Functional neurological disorder (FND)

Functional neurological disorder is a syndrome of involuntary motor, sensory, or cognitive symptoms that vary across contexts. Its presentation distinguishes them from structural damage to the nervous system and instead indicates pathological alterations in the functional organization of brain networks. Following studies in the US and Europe, FND is considered a frequent presentation in neurology clinics, second only to headache, and is likely under-reported.^45^ FND as a broad syndrome has received only limited recognition in Latin America, and we were not able to locate any studies estimating its prevalence in the clinical settings or assessing recognition among clinicians. For specific FND subtypes, functional seizures have been studied more systematically. Functional seizures, sometimes called dissociative or psychogenic non-epileptic seizures, are any combination of altered movement, sensation, or awareness that bears resemblance to epileptic seizures but are not accompanied by epileptiform electrical discharges on electroencephalogram (EEG). A survey of clinicians across 17 countries in Latin America found that psychogenic non-epileptic seizures were a widely recognised diagnosis but deficiencies were identified in training, diagnosis and treatment, including poor healthcare system support with limited access to video-EEG and psychological interventions.^46^ Comparative studies with the US have indicated that Brazilian patients with psychogenic non-epileptic seizures have earlier onset, greater delay in diagnosis, and fewer seizure types,^47^ while patients in Chile were much more likely to be diagnosed based on history and clinical interview alone.^48^ Studies from Argentina,^49^ Brazil,^50^ Colombia,^51^ and Puerto Rico^52^ found similar rates of trauma, psychiatric comorbidity, and proportion of female patients (approximately 70%) to US and European studies. Additionally, a large study in Bolivia reported a strong association between functional seizures and intimate partner violence in women.^53^ Functional seizures can occur alongside epileptic seizures. In studies from Argentina, psychiatric co-morbidity was found to be higher in those with psychogenic non-epileptic seizures compared to epilepsy,^54^ and in those with psychogenic non-epileptic seizures alone when compared to those with comorbid epilepsy.^55^

### Infectious disease and environmental health risks

Latin America has a specific profile of endemic but neglected tropical diseases (NTDs) including trichuriasis, hookworm, Chagas, dengue, malaria, and yellow fever, among others.^56^ Psychiatric and neurological complications of NTDs are now recognised as a major contributor to morbidity via several causal pathways.^57^ These include direct infection of the brain or meninges (e.g. with chagas or neurocysticercosis), secondary impact on the brain after systemic pathology (e.g. stroke due to vascular obstruction caused by lymphatic filariasis), neurodevelopment impairment (e.g. microcephaly after maternal Zika virus infection or impaired cognitive development through childhood hookworm infection), and social stigma and marginalisation—often exacerbated if visible disfigurements or disabilities are caused by the infection (such as with leishmaniasis and leprosy). Although when considered globally, Latin America has a relatively low prevalence of HIV infection, it has the highest global prevalence of HIV-associated neurocognitive disorder^58^ alongside the third highest burden of HIV-associated cryptococcal meningitis.^59^ Although more concentrated, other environmental causes of neuropsychiatric disorders are characteristic of the region and are major contributor to morbidity in specific areas. Tropical Latin America has one of the highest incidence of snakebite envenomation globally which cause high rates of neuropsychiatric morbidity in survivors due to direct neurotoxic effects, psychological trauma, and secondary effects of disability and morbidity.^60^ Large-scale illegal gold mining has become an increasing focus for organised crime networks and exposes workers and community members to mercury poisoning, causing marked neuropsychiatric complications. As a result, populations residing in the Amazon have the world's highest exposure to environmental mercury and mercury-related neurological symptoms.^61^

### Drug and alcohol use

In global terms, Latin America has only a moderate burden of disorders arising from alcohol and illicit drug use.^62^ Nevertheless, these remain an important source of neuropsychiatric problems, and some substances that are more common in the region have specific neuropsychiatric profiles. Cocaine paste (known as *paco* or *basuco*) is a cheap by-product from cocaine production with a high prevalence of use among individuals from marginalised communities.^63^ Compared to powder cocaine, it is associated with increased psychiatric morbidity and higher levels of neuropsychological impairment.^64^ Solvent inhalation is particularly associated with use among people experiencing homelessness in Latin America, likely due to its low cost, easy accessibility, and dual effects of euphoria and appetite suppression.^65^ Although specific inhalants have different clinical profiles, inhalant users have high levels of multi-organ toxicity, increased psychiatric comorbidity, and an acute intoxication syndrome that involves seizures, delirium and cardiac arrhythmia.^66^

## Structural challenges for neuropsychiatry in Latin America

Latin America faces high levels of social challenges that are, in themselves, established causes for psychiatric disorders and may also serve as additive risk factors for those affected by neuropathologies. Poverty, lack of occupational opportunities, armed conflict, political violence, internal displacement, and migration contribute to population mobility and are associated with elevated rates of neurological and psychiatric co-morbidity.^67^ The region has the highest rates of income and health inequality in the world, the latter due to both economic and geographic barriers to access.^68^ Over 30% of the population of Latin America live in poverty with Indigenous and African descendant people experiencing particularly high levels of social marginalisation and poor access to healthcare.^69^ Indigenous peoples of Latin America have strikingly high suicide rates compared to country averages, with marginalisation, industrialisation, environmental degradation, and alcohol misuse being cited as key causes.^70^ Although declining in incidence across the region, recent estimates suggest Latin American women have a 30% prevalence of lifetime physical and/or sexual abuse from an intimate partner.^71^ In one study from Colombia, 31% of women sustained at least one brain injury during an abusive relationship.^72^

Knowledge and recognition of neuropsychiatric disorders in Latin America remains poor with high levels of stigma for mental health and substance use problems.^73^ Awareness and stigma related to neurological disorders have been less extensively studied, but available evidence indicates high levels of stigma for epilepsy in Mexico^74^ and both epilepsy and dementia in Brazil.^75^^,^^76^ Low levels of public knowledge have been cited as barriers for timely care for TBI,^77^ stroke^78^ and epilepsy^79^ in the region. Latin America has a number of folk or cultural concepts of illness (e.g. *paje*, *susto*, and *ataque de nervios*) that do not entirely align with formal diagnostic categories but may be important illness concepts within the communities where they are used.^80^

The needs and barriers for neuropsychiatry professionals in Latin America have been widely discussed in the scientific literature^81^, ^82^, ^83^ and include training, evidence to inform healthcare decisions, collaboration between disciplines, greater participation of stakeholders, and regionally-tailored initiatives. In terms of diagnostic assessment, access to medical technologies like neuroimaging and important diagnostic assays like cerebrospinal fluid analysis are patchy across the region, and often concentrated in urban centres with little infrastructure to facilitate access to rural regions and isolated communities.^84^ Community care is an important model for promoting equitable access to prevention and treatment for remote populations. However, health professionals also remain disproportionately concentrated in urban centres. This creates logistical challenges in ensuring consistent presence, as well as cultural considerations in balancing folk concepts of illness with communicating the need for care.^85^

Assessment of cognitive function is a central component of neuropsychiatric practice due to its role in diagnosis, case formulation, and rehabilitation. Latin America has a strong tradition of neuropsychology and has established training programmes and professional networks across the region. Nevertheless, a recent survey of Latin American neuropsychologists reported that the most frequently mentioned challenges were ‘a lack of normative data from my own country’, assessments ‘not adapted to my culture’, assessments being ‘too costly’ and assessments ‘aimed at individuals with high levels of education’.^86^ This reflects a wider issue of adequately serving the linguistic, cultural, and educational diversity of the region.

## Future directions for Latin American neuropsychiatry

Due to the high prevalence of risk factors for both mental and neurological health issues, neuropsychiatric conditions are a significant concern within Latin America.^87^ Given this context, along with the uneven distribution of healthcare and training resources across the region, it would be unhelpful to view neuropsychiatry as a narrow speciality, restricted to specific professions, and subject to burdensome gatekeeping. Instead, we argue that Latin American neuropsychiatry should be a multi-professional, cross-disciplinary area of research and practice that promotes better understanding and management of neuropsychiatric conditions that bridges traditional service and professional divides.

### Research

Funding for research is scarce in Latin America at almost all levels, from programme grants to post-graduate study and fellowships for early career researchers. An interview-based study with researchers across thirteen Latin American countries found that funding was frequently lacking, limited to small university-level grants, or provided at the researchers’ own expense, with few project grants available.^88^ International funding for research often comes from providers who prioritise diseases more common in Europe and the US, making it more difficult for Latin American researchers to conduct funded research on regional health priorities. Given the unique profile of neuropsychiatric problems in Latin America and their substantial burden, increased funding for research is clearly a priority for the region.

We note that funding Latin American neuropsychiatry research is likely to be of benefit to people beyond its own substantial population. For example, climate change is causing neglected topical diseases to be increasingly common outside traditionally ‘tropical’ regions, meaning the neuropsychiatric impact of, for instance, dengue or neurocysticercosis is a problem that will be increasingly faced in Northern regions of the globe. Latin America has sustained the world's highest rates of violence for decades, and the complex neuropsychiatric consequences of armed conflict, political, and community violence will be faced by other regions as these problems fluctuate around the globe.

However, several challenges must be addressed to strengthen research capacity in the region. While funding is undoubtedly important, valuable contributions are often made by research-trained clinicians working with limited budgets. Additionally, scalable research training is needed to help disseminate best practices from specialist centres to less-resourced areas and countries. Similarly, initiatives to address the ‘opportunity gap’ in neuropsychiatry research are essential to widen participation, which can range from inclusive hiring and mentorship to promoting open research practices and open datasets, all of which serve to reduce barriers to participation and increase the impact of research. A study with mental health researchers in the region found that most international collaborations involved researchers from a single Latin American country working with counterparts in high-income countries, rather than collaborations between Latin American researchers themselves.^81^ Five countries (Argentina, Brazil, Chile, Colombia and Mexico) account for the vast majority of neuroscience research in the region.^89^ Consequently, networks to promote intra-regional collaboration, support, and the development of a neuropsychiatry community should be a priority for the region, in parallel with promoting interest in the region from professional organisations across the world. Good practice can be drawn from areas where Latin America has already developed internationally pioneering research programmes, including in genetic and degenerative disorders.^89^

### Clinical practice and training

There are only a handful of specialist neuropsychiatry training programmes in the region^90^ and neuropsychiatry content varies considerably in standard training programmes for neurologists, nurses, psychiatrists, psychologists and other allied health professions. Specialist centres and clinics are drivers of innovation and best practice and are equally as needed in Latin America. They are not, however, able to provide in-person training in sufficient quantities for the needs of the region. Consequently, a tiered model is needed. High-priority neuropsychiatric topics could be reliably incorporated into mainstream professional training. Accessible online training should be made available for those wanting more depth. Finally, taught courses with mentored clinical rotations available in national or sub-regional hubs is needed to produce highly specialist clinicians.

We note, however, that exactly how neuropsychiatric practice and training fits within existing healthcare and professional structures is not something that could, or should, be determined for Latin America as a whole. The needs of the local populations, the extent to which psychiatric and neurological services overlap and coordinate, and the availability of healthcare resources, will influence how neuropsychiatric practice is integrated into existing structures at the level of service delivery. Nevertheless, better regional coordination can ensure best practice, even for local problems, and should be shared across areas with similar challenges. We also note the importance of making neuropsychiatric training available not just to medical professionals but also for those working in psychology, psychotherapy, physiotherapy and other allied health professions, as each are essential for care. Here, ensuring training resources are available in Spanish and Portuguese is essential, as the English-language format of many current resources is a barrier to access. We also argue for collaboration with lay practitioners such as traditional healers, religious leaders, and community support organisations due to the fact they are often the first point of contact for affected people.

There must be a focus beyond providing improved treatment provision as the sole solution to neuropsychiatric needs in Latin America. Public health approaches that reduce drug and alcohol use, infectious disease vectors, violence, abuse, industrial and traffic accidents, dementia, and cardiovascular risk factors, are all likely to have an important impact on the burden of neuropsychiatric disorders. Therefore, coordinated efforts between medical and public health actors are likely to be key, improving public knowledge about neuropsychiatric problems in culturally sensitive and appropriate formats alongside evidence-based policy changes. We note that public health initiatives will likely be unevenly relevant within Latin America due to its considerable diversity. While some initiatives may be relevant internationally, some may be best developed at the level of population, community or even ecoregion. Given that the family is the most common and often, the most effective, source of neuropsychiatric care in Latin America, specific forms of culturally appropriate family education and support are likely to be of huge benefit. Indeed, patients, families and communities should not solely be the recipients of neuropsychiatry but should be included as active participants in the development of neuropsychiatric services that are best placed to serve their needs. Latin American models of recovery that focus on meaningful improvement for affected individuals informed by patient priorities, local culture, and region-specific barriers to maintaining good mental and neurological health are essential to progress.

## Conclusions

There is a danger in arguing for a move ‘towards a Latin American neuropsychiatry’ of appearing to suggest that there is currently little neuropsychiatry practiced in Latin America. This is clearly not the case, as the many examples in this article show. Instead, we are advocating for something that is much needed but under-discussed: the development of an explicitly Latin American neuropsychiatry, drawing from and focused on challenges in the region, developed into an open, cohesive community of practice. Given the shared concerns of the region, the need for region-specific evidence, and a currently fertile ground for improvements in regional collaboration, research, and clinical practice, we argue that moving towards an explicitly Latin American neuropsychiatry has much to gain for people in the region and much to contribute worldwide.

## Contributors

This article was conceptualised by JRB and VB. It was drafted by JRB and VB. All authors were involved in the review and editing of the final version.

## Editor's note

The Lancet Group takes a neutral position with respect to territorial claims in published maps and institutional affiliations.

## Declaration of interests

AS has received travel grants from the Global Brain Health Institute and IMPACT Dementia project (NIHR150287) and is on the advisory board for both of these grant-funded projects. VB is an unpaid committee member of the British Neuropsychiatry Society (Charity No. 1154725). All other authors have no interests to declare.

## Appendix A Supplementary data

Translated summary
