# Supplementary material for: Towards a Latin American neuropsychiatry: challenges and opportunities
Source: Lancet Reg Health Am. 2025 Dec 2;54:101322. doi: 10.1016/j.lana.2025.101322 (PMC12719751; doi:10.1016/j.lana.2025.101322)
Supplement: Translated summary [file mmc1.pdf]

__Editorial disclaimer:__ These translations in Spanish and Portuguese were submitted by the authors, and we reproduce them as supplied. It has not been peer reviewed. Our editorial processes have only been applied to the original abstract in English, which should serve as reference for this manuscript.

Translated summaries for: Towards a Latin American Neuropsychiatry: challenges and opportunities

Español

Desde el impacto del conflicto armado y la violencia política, hasta las consecuencias neuropsiquiátricas de las enfermedades tropicales desatendidas, América Latina tiene un perfil único de factores de riesgo específicos de la región que significa que no siempre está bien atendida por la práctica neuropsiquiátrica desarrollada en las regiones de altos ingresos. Aquí, revisamos las características neuropsiquiátricas específicas de la región de lesiones cerebrales traumáticas, accidentes cerebrovasculares, epilepsia, demencia, trastornos neurológicos funcionales, enfermedades infecciosas, riesgos ambientales para la salud y el consumo de sustancias. Además, identificamos desafíos estructurales para la salud neuropsiquiátrica y sugerimos caminos para desarrollar una neuropsiquiatría específicamente latinoamericana como un campo multidisciplinario y multiprofesional basado en pasos prácticos para fortalecer la capacidad de investigación, la formación, la práctica clínica y la entrega de atención. América Latina debería ser una prioridad para la neuropsiquiatría y defendemos una neuropsiquiatría latinoamericana que tiene mucho que ofrecer a la región y mucho que aportar a nivel mundial.

Português

Desde o impacto dos conflitos armados e da violência política até as consequências neuropsiquiátricas das doenças tropicais negligenciadas, a América Latina possui um perfil único de fatores de risco específicos da região, o que significa que nem sempre é bem atendida pela prática neuropsiquiátrica desenvolvida nas regiões de alta renda. Aqui, revisamos as características neuropsiquiátricas específicas da região em relação a lesões cerebrais traumáticas, acidente vascular cerebral, epilepsia, demência, transtorno neurológico funcional, doenças infecciosas, riscos ambientais à saúde e uso de substâncias. Além disso, identificamos desafios estruturais para a saúde neuropsiquiátrica e sugerimos caminhos para desenvolver uma neuropsiquiatria especificamente latino-americana como um campo interdisciplinar e multiprofissional, com base em passos práticos para fortalecer a capacidade de pesquisa, a formação, a prática clínica e a entrega de cuidados. A América Latina deve ser uma prioridade para a neuropsiquiatria e defendemos uma neuropsiquiatria latino-americana que tem muito a oferecer à região e muito a contribuir mundialmente.
